# Experiences of mental healthcare providers regarding integration of mental healthcare into primary healthcare at the iLembe health district in KwaZulu-Natal province

**DOI:** 10.4102/hsag.v25i0.1143

**Published:** 2020-05-04

**Authors:** Siphiwe T. Madlala, Respect M. Miya, Mdumiseni Zuma

**Affiliations:** 1 Department of Nursing Science, University of Zululand, Empangeni, South Africa

**Keywords:** integration, mental healthcare, primary healthcare, professional nurse, mental healthcare-user

## Abstract

**Background:**

Institutionalisation of mental healthcare users was a prevalent treatment approach in the apartheid era in South Africa. The introduction of community-based mental healthcare is aimed at improving mental healthcare services. The integration into primary healthcare improves access to mental health services. This integration is implemented by professional nurses working in the primary healthcare clinics.

**Aim:**

The aim of this study was to explore and describe experiences of professional nurses regarding integration of mental health into primary healthcare at the iLembe district of KwaZulu-Natal.

**Setting:**

This study was conducted at a public hospital in the iLembe health district of KwaZulu-Natal province.

**Methods:**

An explorative, descriptive and contextual qualitative research study was conducted. Semi-structured interviews were conducted with 15 professional nurses. Data were transcribed verbatim, organised into codes and finally analysed thematically using Tesch’s method of data analysis.

**Results:**

This study revealed that majority of participants experienced and faced challenges regarding integration of mental health into primary healthcare. Professional nurses stated that they require support from the management and training to equip themselves with knowledge and skills to render effective mental healthcare services.

**Conclusion:**

The integration of mental healthcare into primary healthcare is pivotal to ensure easy and accessible mental healthcare services to its users. This strategy requires planning and implementations of available policies and guidelines regarding mental healthcare. Furthermore, adequate funding is required to secure human and material resources to aid the integration of mental healthcare into primary healthcare.

## Introduction

Institutionalisation of mental healthcare users served as the primary and most dominant treatment approach before and during the apartheid era in South Africa (Lucas & Stevenson 2006). As one of a number of evidence-driven service improvement modifications, the country’s Department of Health prioritised improvement in mental healthcare by recommending, *inter alia*, deinstitutionalisation and reintegration of mental healthcare users into the community (Skeen et al. 2010).

These improvements coincided with South Africa’s political independence. Mental healthcare services remained a seminal challenge in most of the healthcare institutions as many mental healthcare users are hospitalised for a long time in institutions. The introduction of community-based mental healthcare is aimed at improving mental healthcare services. Therefore, integration of mental healthcare services into primary healthcare has improved access and enabled mental healthcare users to maintain contact with their families and to remain employed whilst receiving treatment and psychosocial rehabilitation.

The implementation of community-based care services has been particularly problematic for African countries because of a number of issues such as poorly developed legislative provision and limited community-based care alternatives. According to Jack-Ide, Uys and Middleton (2012), Nigeria, one of Africa’s largest nations, lacks legislation compared to other countries. By comparison, the South African mental health system has a number of characteristics that offer advantages, and these include the fact that the country already has some resources such as facilities for psychotropic medications and outreach clinics – all of which are central requirements for providing mental healthcare. The National Mental Health Policy in South Africa promotes deinstitutionalisation by treating mental health as an integral part of primary healthcare (World Health Organisation [WHO] 2014). This promotes short hospital stays whilst encouraging the use of primary healthcare services. Nevertheless, evidences of discharge of long-term mental healthcare patients from hospitals into the community have not matched national and international drive towards deinstitutionalisation (Krüger & Lewis 2011).

The available facilities do not have the expertise to care for more severely disabled and disturbed mental healthcare users (Krüger & Lewis 2011).

In KwaZulu-Natal, the implementation of *Mental Health Act* 17 of 2002 (Republic of South Africa 2002) has improved access to mental healthcare services in the province, although significant gaps, such as infrastructure, staffing, training and administrative requirements, need to be addressed before the implementation is deemed a success (Ramlall, Chipps & Mars 2010). The World Health Organization (2014) maintains that the principles of community-based services must be in place. To be in line with the WHO, former institutional residents need access to mental health services, including evidence-based clinical care, and also access to social services for help with housing, employment and community reintegration. Staff shortage in primary healthcare clinics is a barrier in providing quality mental healthcare, especially in rural clinics, because these clinics are overburdened with multiple programmes and high patient load (Dube & Uys 2015).

## Problem statement

The integration of mental healthcare into primary healthcare is the approach implemented worldwide, primarily as a means of ensuring the provision of effective community-based care for affected individuals (WHO 2010).

A number of challenges are encountered in this approach, but studies have identified and recommended the solutions as well. These challenges range from lack of mental healthcare facilities to problems of distance travelled to access mental healthcare facilities, amongst others. Hence, the integration of mental healthcare services into primary healthcare services is vital. The treatment rates for mental disorders are globally low and reveal a worrisome picture where treatment rates range from 13% to 33% in high-income countries and from 5% to 13% in low- and middle-income countries (WHO 2014).

A limited range of studies have been conducted around the concept of integration of mental healthcare services into primary healthcare and the benefits and challenges faced by the phenomenon of mental healthcare. This study was conducted to fulfil this gap as little was known about the experiences of South African professional nurses rendering mental healthcare services, and the integration of mental healthcare into primary healthcare facilities from South African context.

## Aim

The aim of this study was to explore and describe experiences of professional nurses regarding integration of mental health into primary healthcare at the iLembe district in KwaZulu-Natal province.

## Research methods and design

### Study design

An explorative and qualitatively descriptive research design was used to conduct the study. An explorative research design seeks to gain insight into a situation, phenomenon and a community (Brink, van der Walt & van Rensburg 2012), whereas a qualitatively descriptive research presents a picture of specific details of a situation and focuses on the deeper meaning and intensive examination of the phenomenon under study (Brink et al. 2012). Therefore, explorative and qualitatively descriptive research design was used to explore and describe experiences of professional nurses regarding integration of mental healthcare into primary healthcare at the iLembe health district in KwaZulu-Natal.

### Study setting

The study was conducted at a public hospital of iLembe health district. According to the Department of Health, KwaZulu-Natal (2015), the selected hospital serves as a referral hospital from its four affiliated clinics and serves a population of 16 0342 from the areas of Kranskop, Ngcolosi, Mabomvini, Makhabeleni, Cele and Mahlongwa (Anon 2016). In KwaZulu-Natal province, the healthcare institutions are categorised according to the services provided. The primary healthcare institutions are the first patient-contact institutions which refer patients to level 1 district hospitals, which refer their patients to level 2 regional hospitals, which, in turn, refer their patients to level 3 tertiary hospitals for more advanced healthcare interventions. In iLembe healthcare district, the level 1 district hospital offering primary healthcare and mental healthcare nursing services is located in Kranskop, a small town in the north of KwaZulu-Natal province. All primary healthcare institutions, including the level 1 district hospital, rendering mental healthcare services at iLembe healthcare district were sampled to be included in the study, and all clinics not rendering mental healthcare facilities in this district were excluded from this research.

### Study population and sampling strategy

According to Brink et al. (2012), population is regarded as an entire group of persons or objects that are of interest to the researcher, the entire set of individuals having some common characteristics. In this study, the study population comprised all professional nurses providing mental healthcare services at all mental healthcare institutions conforming to the inclusion criteria of the study. Professional nurses working with mental healthcare users at both primary healthcare centres and level 1 district hospitals were purposefully selected for inclusion. This was done because of their duties regarding mental and primary healthcare such as reviewing, assessing and prescribing medication and implementation of outreach campaigns. The study sampling size was determined by data saturation, which was reached after 15 semi-structured interviews with participants. An additional two semi-structured interviews were conducted with participants to confirm final data saturation, which was also added in data analysis of this study.

### Data collection

The researcher developed the data collection tool with the supervision of two study supervisors having doctorate degrees. The data collection tool was pre-tested with three participants for its reliability, and no changes were found necessary to be further affected on its usage. Neither the data collected during pre-testing phase nor the participants were included in the main study. Data were collected through individual semi-structured interviews with selected professional nurses, lasting for 30 min to an hour, depending upon the information obtained from participants. Semi-structured interviews were done in English and assisted in gaining in-depth knowledge, as questions were clarified where necessary using probing and follow-up questions (Brink et al. 2012). The interview sessions were audio-recorded and field notes were also taken.

Fifteen interviews with participants were conducted before data became saturated. This was followed by additional five interviews to ensure that data saturation was reached. The data collection was done for over 15 days during March 2018 depending on the availability of participants.

### Data analysis

Data analysis was done concurrently with data collection (Brink et al. 2012) using Tesch’s method of data analysis for qualitative research (Tesch’s method as cited in Creswell 2014). Tesch’s eight steps of data analysis were applied that included reading through all transcripts, jotting down thoughts, listening through the voice recorder and finding common themes and sub-themes. The primary researcher M.Z. transcribed data verbatim, which was coded to ensure confidentiality. Common themes and sub-themes that emerged during data analysis were captured. The transcribed data and the audio recordings were examined by two study supervisors, S.T. and R.M., for confirmability. Once audio recordings and transcribed data were examined by the supervisors, they were handed over to an independent examiner holding a doctorate degree to ensure credibility. The independent examiner signed a declaration form before examining the data to ensure confidentiality. The points on which the researcher, supervisors and examiner disagreed were further discussed to reach at an agreement by all stakeholders. Dependability was ensured by safeguarding that each process in the study was reported in detail so that an external researcher could repeat inquiry to achieve similar results. An audit trail of all steps taken in reaching decisions during analysis was kept to be used for purpose of transferability (Polit & Beck 2008).

### Ethical considerations

Ethical clearance was obtained from the University of Zululand Research Ethics Committee (UZREC), and permission to conduct the study was obtained from the KwaZulu-Natal Department of Health Research Unit as well as from the iLembe health district manager. After permission was granted by gatekeepers, the researcher approached participants. The information letter and consent form were given to all participants. To address the ethical issues related to participants, three fundamental ethical principles were taken care of throughout the research process: respect of persons, beneficence and justice (Brink et al. 2012). Complete information about the study was given to all participants, including the aim, benefits and risks associated with the study to ensure their respectability; beneficence was ensured so that the study bore no harm and financial burdens on participants. To ensure justice, all information provided by the participants was treated confidentially by using codes (Brink et al. 2012). All transcripts and the audio recorder used were kept by the researchers under lock and key and softcopies were password-protected to ensure confidentiality.

## Findings of the study

During data collection and analysis, a number of themes and sub-themes emerged, which were discussed based on the study’s objective with supporting statements as given in Table 1.

**TABLE 1:** Themes, sub-themes and supporting statements.

Themes	Sub-themes	Statements
Administration	Staffing of professional nurses The system for the management of referrals	‘The mental healthcare users are more than the staff ration in this institutions.’ Participant 7 ‘There is no clear referral system between the institutions when it comes to the mental healthcare users, this causes a lot of confusion.’ Participant 12
Resources	Challenges related to availability and proper allocation of funds Challenges related to availability of working material (medication)	‘Mental healthcare is not prioritised when budget is done as we are struggling to have sufficient resources to do community outreach and to attend training as professional nurses.’ Participant 3 ‘We do not have enough medication to give to the mental healthcare users.’ Participant 8
Knowledge and skills	Guidelines and policies Training Areas related to scope of practice	‘There are policies and guidelines available but they are not effectively implemented at this institutions.’ Participant 2 ‘We are not being sent to in-service training nor to do advance psychiatric nursing to improve our knowledge.’ Participant 13 ‘The Department always tells us that there are no funds.’ Participant 15 ‘We do all the work here even those that are above our scope of practice.’ Participant 5
Rendering of care	Poor service delivery	‘I feel that the service we are rendering to the clients is not of quality standard due to lack of resources, this affects them negatively towards their progress.’ Participant 14 ‘We are feeling stressed about the sub-standard services we are forced to provide to our clients. This exposes us to end up with litigations.’ Participant 10 ‘I am not comfortable about the level of my service I am providing to the clients.’ Participant 7
Influence on the care of mental healthcare users	Mental healthcare users relapse Early discharge of mental healthcare users in hospitals	‘Lack of managing the process of integration and support of staff can contribute to relapsing of the clients.’ Participant 4 ‘The clients are being discharged not being ready to face the outside world.’ Participant 9

## Discussion on findings

The study findings were discussed based on the emerged themes and sub-themes.

### Administration

#### Staffing of professional nurses

In most mental healthcare institutions, there is a lack of available professional nurses trained to implement the integration of mental healthcare into primary healthcare. The participants alluded that there is a great shortage of mental healthcare-trained professional nurses in iLembe health district. World Health Organization (2016) revealed that in some countries, primary care staff is already overburdened with work, and integration of mental healthcare into primary healthcare would require an increase in the absolute number of primary healthcare staff. There is a need to have sufficient staff trained in psychiatry, with knowledge and authority to prescribe psychotropic medicines at primary and secondary levels (WHO 2016). The reasons raised by participants included shortage of personnel, which was associated with unemployment and slow training process of professional nurses. Dube and Uys (2015) agree with this notion by stating that shortage of staff in primary healthcare clinics is seen as a barrier towards the integration of mental healthcare into primary healthcare and provision of quality mental healthcare, especially in rural clinics because these clinics were already burdened with multiple programmes and high patient load.

The study confirmed the findings of the WHO (2014) which stated that shortage of professional nurses for mental healthcare users has become a global phenomenon. According to the Department of Health, Republic of South Africa (RSA; 2013), there is a gradual decline in the number of nurses with specialised qualifications such as critical care, child care, operating theatre, advanced midwifery and advanced psychiatry nursing (Department of Health, RSA 2013). Hence, the quality of mental healthcare services is declining which is detrimental for mental healthcare users. Decline in the number of nurses with specialised training in advanced psychiatry contributed to the poor and slow integration of mental healthcare into primary healthcare based on the ratio of available staff versus the number of mental healthcare users. The WHO (2014) suggested that the governments must pay attention to key human resource management issues in primary healthcare, adequate working conditions, payment, resources and support to carry out demanding work to integrate mental healthcare into primary healthcare.

#### Referral system

Participants were of the view that current referral system caused the influx of mental healthcare users to clinics. They felt that services were unclear in their admission criteria and this often led to overcrowding by mental healthcare users at the clinics. The WHO (2007) revealed that the absence of a good referral system between primary and secondary care severely undermined the effectiveness of mental healthcare delivered at primary healthcare level. Furthermore, most participants alluded that it was because of the system was not functioning well or proper referral procedures were not followed. According to Department of Health, RSA (2007), proper criteria for referral of mental healthcare users should be established and followed by all stakeholders. This is further supported by Hattingh and Joubert (2019), who stated that clients must access mental health services closer to their homes (thus avoiding costs associated with seeking specialist care at distant locations).This would minimise stigmatisation and discrimination associated with mental health services delivered at primary care and ensure holistic treatment that addresses both physical and mental healthcare needs.

The participants stated that poor referal system by the hospitals leads to the referal of unstable mental healthcare users to primary healthcare. This leads to relapse of treatment in many mental healthcare users which contributes to poor integration of mental healthcare into primary healthcare. Hattingh and Joubert (2019) stated that referral to a primary healthcare was generally viewed as something negative that could even adversely affect patient’s mental health, for example, by relapsing or turning to substances for relief. Therefore, it is imperative to have clear guidelines on how mental healthcare users are referred from one institution to another, specifically to primary healthcare centres.

### Resources

#### Availability and proper allocation of funds

Most of the participants were of the view that mental health is not prioritised during allocation of funds.

Currently, South Africa spends 5% of its health budget on mental health, but evaluations of economic burden of mental illness and cost effectiveness of service scale-up indicate that the total costs of untreated mental illness far outweigh the proposed treatment costs (WHO 2011). This was also confirmed by Abdelgadir (2012), who stated that globally there is inadequate financial support for mental healthcare programmes. The implementation of the integration of mental healthcare into primary healthcare requires adequate funds to ensure availability of adequate material and human resources, including properly planned infrastructure. Trends in mental health show that public funds for the sector have been historically low and available funds are also not properly utilised at times. For instance, funds allocation to mental healthcare is less than 1% of the total health expenditure (Ashadeep 2012). These lead to problems in the implementation of integration of mental healthcare into primary healthcare. The WHO (2007) report has suggested that funds must be shifted or redistributed from tertiary to secondary and primary levels of care, including the community-oriented mental healthcare services, or new funds must be made available. This would assist in the integration of mental healthcare into primary healthcare.

#### Availability of working material (medication)

The study findings revealed that professional nurses find it difficult to perform their duties because of either limited or non-availability of working material. Mental healthcare users are required to be under medication to remain stable and to minimise the likelihood of inflicting harm to themselves or to the community. Basic psychotropic medicines must be made available at primary and secondary care levels. Therefore, the governments have to ensure that sufficient funds are allocated to purchase the basic essential psychotropic medicines and make sure that they are available at primary care settings (WHO 2007). The South African Primary Healthcare Level Standard Treatment Guidelines and Essential Medicines List make provision for a limited range of psychiatric drugs for primary healthcare centres (Hattingh & Joubert 2019). Professional nurses should be supported by management with adequate resources such as medicines to be issued to mental healthcare patients. This would promote adherence to prescribed psychotropic treatment and reduce chances of relapse of mental healthcare users because of unavailability of prescribed medication at primary healthcare.

#### Guidelines and policies

The *South Africa Mental Healthcare Act* 17 of 2002 (Republic of South Africa 2002) states that mental healthcare should be fully integrated into primary healthcare, and mental healthcare practitioners should take responsibility of needs of mental healthcare. This should be done by implementing the guidelines and policies available in the healthcare sector regarding the phenomenon. Mental Health and Poverty Project (2008) revealed various barriers regarding the implementation of mental healthcare guidelines and policies such as insufficient lobbying and technical support from the national office for implementation of legislation in provinces; limited human resources for implementation of policy and legislation because of high turnover of clinical staff and/or inadequate training in the implementation of policy and legislation; and insufficient promotion of advocacy and activism within communities and the mental health regarding the implementation of mental health policy and legislation. Furthermore, participants were of the view that even though policies and guidelines were available to guide practice, they were not followed because of overburden of primary healthcare with multiple programmes required to be implemented by limited staff. Failure to follow set guidelines and policies often leads to serious negative outcomes in mental healthcare services, including, amongst others, relapse of mental healthcare users from treatment (Mental Health and Poverty Project 2008).

#### Training

Most of the participants reported that they were not provided regular training and had outdated knowledge of mental healthcare. According to the participants this was due to general lack of funds from the Department of Health. Delivery of mental healthcare within primary healthcare systems requires a radical role transition of healthcare professionals, mainly from service delivery to programme design, training, supervision, consultation–liaison for complex cases and evaluation of programmes at primary healthcare level and in the community (Patel 2013). This was further supported by the Department of Health (WHO 2007) which indicates that basic awareness training, training in safe functioning, support to identify role boundaries, education about cultural issues and information about local services are as important as training in specific skills. The training of professional nurses working at primary healthcare is essential to equip them with current knowledge and skills regarding how to treat mental healthcare users, including the integration of mental healthcare into primary healthcare.

#### Scope of practice

Participants raised a concern that because of shortage of psychiatrists and advanced mental health nurses, they were often being forced to perform duties outside their legislated scope of practice, thus putting their careers and lives of mental healthcare users at risk. Practising outside one’s scope of practice exposes healthcare providers and mental healthcare users at risk. All mental healthcare providers are given scope of practice under which they are expected to practise (South African Nursing Council [SANC] 1991). Failure to practise within the set SANC scope of practice jeopardises careers of professional nurses, leading to contravening of *Nursing Act* 22 of 2005.

### Rendering of care

#### Poor service delivery

Poor service delivery, that is, service below expected standards, leads to stress in mental health providers; this may contribute to negative attitudes towards mental health and an increase in litigations. The Department of Health, RSA (2013) has admitted that there is an increased number of litigations against the department. During interviews, the participants emphasised their lack of confidence in the services they are rendering to the community. Barriers to the fostering of a task-sharing approach include significantly poor rate of staff turnover in many primary healthcare settings, requirement of substantial training and supervision, and burdening of primary healthcare workers with tasks they cannot perform reasonably well (Ventevogel 2014). Services below expected standards lead to stress in professional nurses and this also leads to mental healthcare patients not getting the services they are entitled to have. The *South Africa Mental Healthcare Act* 17 of 2002 (RSA 2002) states that mental healthcare should be fully integrated into primary healthcare, and mental healthcare practitioners should take responsibility of providing mental healthcare services to the patients with mental disorders. The WHO (2007) has emphasised that to render quality mental healthcare services, professional nurses must have the knowledge, skills and motivation to treat and manage patients suffering from mental disorders.

### Influence on the care of mental healthcare users

#### Mental healthcare users relapse

Poor planning and implementation of integration process of mental healthcare into primary healthcare could have negative consequences for mental healthcare users. These consequences could include, amongst others, relapse from treatment of such patients. The mental healthcare requires public health sector to ensure that primary healthcare must have adequate infrastructure, staff, expertise and resources to provide necessary care for mental patients (Hattingh & Joubert 2019). Failure of primary healthcare service providers to render adequate and efficient care to mental patients may lead to absence of patients from scheduled appointments, and not collecting and adhering to their prescribed psychotropic treatment. According to Patel et al. (2013), mental healthcare users suffering from mental disorders, such as depression, anxiety and alcohol addiction, must not be rendered specialised treatment by non-specialists in healthcare settings because such patients tend to relapse if they are not treated in organised and structured healthcare institutions. Furthermore, primary healthcare staff have to be adequately supervised, monitored and supported by mental health specialists (professional and secondary levels) if integration is to succeed (WHO 2007). Moreover, the mental health professionals should discuss difficulties with management and provide advice on interventions to be carried out by primary healthcare staff to prevent patients relapsing from their treatment.

#### Early discharge of mental healthcare users in hospitals

The simple meaning of mental health integration into primary healthcare is to treat mental healthcare users in community-based facilities near their homes. The participants reported that hospitals were inappropriately taking advantage of such integration of mental healthcare into primary healthcare by discharging mental healthcare users to clinics and community when little or no after-care was arranged. The Mental Health Chief Psychiatrist Guidelines (Department of Health & Human Services, 2002) suggest that mental health hospital providing a clinical service should be prepared to work on discharge planning with their patients. Furthermore, regular clinical review and case load audits should consider the required level of service, who should provide such service and whether alternative service providers are available in primary healthcare prior to discharging of mental healthcare users to avoid relapses (Department of Health & Human Services, 2002). This would lead to proper assessment of institutionalised mental healthcare user’s readiness to be discharged and referred to primary healthcare institution for further treatment after proper consultation and referral system to this primary healthcare institution.

### Recommendations

- The administrators overseeing the integration of mental healthcare into primary healthcare should consider increasing human resources specialised in advanced psychiatric nursing at primary healthcare institutions to ensure proper integration and provision of quality mental healthcare services.
- The administrators should support professional nurses assigned to implement the integration of mental healthcare into primary healthcare.
- There should be policies and guidelines available on how mental healthcare users are referred from mental healthcare hospitals to primary healthcare, and these must be interpreted and implemented correctly to avoid confusion.
- Proper communication between referring and referred institutions should be maintained prior to discharge of mental healthcare user to ensure continuity of care.
- The administrators should consider allocating adequate funds to ensure that the process of integrating mental healthcare into primary healthcare is funded adequately to cover expenditures on resources and infrastructure, including availability of medications.
- Professional nurses and other stakeholders involved in the integration of mental healthcare into primary healthcare should implement available policies and guidelines.
- Regular in-service training on the care of mental healthcare patients at primary healthcare institutions should be held for professional nurses rendering such services.
- Professional nurses working at primary healthcare not having advance training in psychiatric nursing as a speciality should be provided such training.
- Professional nurses rendering mental healthcare should work within their scope of practice to avoid litigations.
- Support should be offered by administrators to professional nurses rendering mental healthcare to ensure that quality of service is provided to such patients.
- The administrators should consider having adequate resources (both human and material), staff supervision and monitoring of the process of integrating mental healthcare into primary healthcare to limit relapse in mental healthcare patients.
- Proper assessment and evaluation of mental healthcare users’ readiness to be shifted from mental healthcare hospitals to primary healthcare should be done by a multidisciplinary health team.

## Study limitations

The study was conducted at a level 1 public hospital and its various clinics situated in iLembe district. If the study was extended to other districts in KwaZulu-Natal province, it would have yielded different findings.

## Conclusion

The integration of mental healthcare into primary healthcare is an initiative aimed at rehabilitating mental healthcare users back into the mainstream of the society. This strategy needs proper planning and implementation with the support of administration to ensure that professional nurses are adequately trained and motivated to implement the integration process. This should include regular holding of in-service training to capacitate professional nurses on how to render services to mental healthcare users at primary healthcare level. Furthermore, proper infrastructure suitable for consultation and holding rehabilitation programmes for mental healthcare users should be available, including enough psychotropic drugs to avoid relapse in mental healthcare users because of unavailability of treatment. Moreover, policies and guidelines should be implemented correctly regarding the referral system and the care rendered by professional nurses to mental healthcare users.
